# Low Primary and Secondary HIV Drug-Resistance after 12 Months of Antiretroviral Therapy in Human Immune-Deficiency Virus Type 1 (HIV-1)-Infected Individuals from Kigali, Rwanda

**DOI:** 10.1371/journal.pone.0064345

**Published:** 2013-08-12

**Authors:** John Rusine, Brenda Asiimwe-Kateera, Janneke van de Wijgert, Kimberly Rachel Boer, Enatha Mukantwali, Etienne Karita, Agnes Gasengayire, Suzanne Jurriaans, Menno de Jong, Pascale Ondoa

**Affiliations:** 1 Amsterdam Institute for Global Health and Development (AIGHD), Department of Global Health, Academic Medical Center, Amsterdam, The Netherlands; 2 National Reference Laboratory, Kigali, Rwanda; 3 Project San Francisco, Kigali, Rwanda; 4 Academic Medical Center, Department of Medical Microbiology, Amsterdam, The Netherlands; 5 University of Liverpool, Institute of Infection and Global Health, Liverpool, United Kingdom; 6 Royal Tropical Institute, Biomedical Research, Amsterdam, The Netherlands; 7 The Infectious Diseases Network for Treatment and Research in Africa (INTERACT) project, Kigali, Rwanda; Boston University, United States of America

## Abstract

Treatment outcomes of HIV patients receiving antiretroviral therapy (ART) in Rwanda are scarcely documented. HIV viral load (VL) and HIV drug-resistance (HIVDR) outcomes at month 12 were determined in a prospective cohort study of antiretroviral–naïve HIV patients initiating first-line therapy in Kigali. Treatment response was monitored clinically and by regular CD4 counts and targeted HIV viral load (VL) to confirm drug failure. VL measurements and HIVDR genotyping were performed retrospectively on baseline and month 12 samples. One hundred and fifty-eight participants who completed their month 12 follow-up visit had VL data available at month 12. Most of them (88%) were virologically suppressed (VL≤1000 copies/mL) but 18 had virological failure (11%), which is in the range of WHO-suggested targets for HIVDR prevention. If only CD4 criteria had been used to classify treatment response, 26% of the participants would have been misclassified as treatment failure. Pre-therapy HIVDR was documented in 4 of 109 participants (3.6%) with an HIVDR genotyping results at baseline. Eight of 12 participants (66.7%) with virological failure and HIVDR genotyping results at month 12 were found to harbor mutation(s), mostly NNRTI resistance mutations, whereas 4 patients had no HIVDR mutations. Almost half (44%) of the participants initiated ART at CD4 count ≤200cell/µl and severe CD4 depletion at baseline (<50 cells/µl) was associated with virological treatment failure (p = 0.008).

Although the findings may not be generalizable to all HIV patients in Rwanda, our data suggest that first-line ART regimen changes are currently not warranted. However, the accumulation of acquired HIVDR mutations in some participants underscores the need to reinforce HIVDR prevention strategies, such as increasing the availability and appropriate use of VL testing to monitor ART response, ensuring high quality adherence counseling, and promoting earlier identification of HIV patients and enrollment into HIV care and treatment programs.

## Introduction

Improved access to combined antiretroviral therapy (ART) has significantly reduced HIV-related morbidity and mortality worldwide [1]. To date, HIV treatment and care programs in sub-Saharan Africa have implemented a public health approach [2] with good access to a limited number of first and second-line ART regimens and CD4 count monitoring, but little attention paid to HIV viral load monitoring and the detection of HIV drug resistance (HIVDR). In 2010, more than five million HIV-infected Africans were estimated to receive life-saving ART, with Rwanda reporting treatment coverage of 80% [3].

However, ART scale up in resource-poor settings could accelerate HIVDR emergence [4], [5], [6], [7] due to insufficient viral load (VL) monitoring [8], inconsistent drug supply [9], and possible unregulated use of antiretroviral drugs (ARV) [10].

HIVDR can develop because of the error prone nature of HIV replication resulting in a high mutation rate in combination with the ongoing presence of drug-selective pressures. HIVDR strains that emerge after treatment initiation (referred to as acquired or secondary HIVDR) can subsequently be transmitted to previously uninfected patients (referred to as transmitted or primary HIVDR) [11], [12]. Transmitted HIVDR increases the risk of virological therapy failure [13] and compromises the efficacy of first-line ART regimens. This is important in a context of limited treatment options.

HIVDR increases direct medical and laboratory costs associated with treatment failure as well as indirect health care costs associated with having to switch patients to more expensive second-line therapy and the ongoing need to develop new drugs. Therefore, HIVDR prevention should be an important public health goal in countries with limited resources.

Recent reports indicate that the lack of VL monitoring during ART leads to late detection of virological failure. Long-term exposure to failing regimens facilitates the emergence and accumulation of acquired HIVDR mutations [8], [14], [15]. The East African region has a high prevalence transmitted HIVDR [16], [17] and prevalence is highest in countries of early ART roll-out and high ART coverage [8], [14], [18] Collectively, these observations underscore the need to monitor HIVDR in countries that are scaling up ART in order to remedy programmatic deficiencies in a timely fashion and protect the efficacy of first and second-line ART regimens. In Rwanda and most other resources-constrained countries, routine VL and HIVDR testing is not available to support routine HIV clinical care due to high costs. Moreover, many patients are diagnosed late and commence ART at lower CD4 counts [19], which increases risk of treatment failure [20], [21].

Since Rwanda initiated ART roll-out in 2004, few data have been generated on treatment outcomes in general, and prevalence and incidence of transmitted and acquired HIVDR in particular. Here, we describe transmitted HIVDR at baseline, and treatment outcomes and acquired HIVDR one year after ART initiation in a cohort of Rwandan HIV patients. Treatment outcomes were specifically examined as a function of immunological status at ART initiation, and other potential determinants of virological failure after 12 months of treatment were also examined.

## Materials and Methods

### Ethics statement

Ethical clearance was obtained from the Rwandan National Ethics Committee. All study participants provided written informed consent prior to study enrolment. Participants had the right to withdraw from the study at any time.

### Study design

The present study was part of a larger prospective investigation addressing the secondary effects of HIV treatment and its impact on reproductive health in a cohort of patients initiating ART (Side effect and Reproductive Health in a Cohort on HAART: SEARCH). The study was conducted at the Treatment and Research AIDS Center (TRAC-Plus) outpatient clinic. Patients were enrolled between November 2007 and January 2010, and were followed up for a maximum of 24 months per person. In this report, only data up to month 12 of follow-up and collected before the 15^th^ of September 2010 were analyzed.

Medical visits were scheduled at baseline (ART initiation), week 2, month 1, month 3 and every three months thereafter until month 12. Information on demographics, sexual behavior, clinical history and physical examination was collected at these visits. Pharmacy visits were scheduled at baseline, every week during the first four weeks of ART (week 1 to 4), and every month thereafter (month 2 to 12). During these visits, medications were delivered and treatment adherence was monitored. Laboratory tests (CD4 count, HIV VL and HIVDR genotyping) were scheduled at baseline, month 6 (CD4 count only) and month 12. Study staff minimized loss-to-follow-up by actively contacting participants who had missed a scheduled clinic visit and by providing travel reimbursements to participants.

### Study participants

HIV-positive patients attending the TRAC-Plus clinic, who were ARV-naive and immediately eligible for ART according to the Rwandan national guidelines [22], were asked to participate in the study. Other inclusion criteria were being 18 years of age or older, residing and planning to reside within travel distance from the TRAC-Plus clinic for the duration of follow-up, willing and able to adhere to the study protocol, and willing and able to give written informed consent for study enrollment. The main exclusion criteria were being pregnant and laboratory or clinical diagnosis or suspicion of tuberculosis. Previous use of ARVs in the context of prevention of mother-to-child transmission was not an exclusion criterion.

### HIV treatment

ART was provided through the national HIV treatment program. Participants received a first-line regimen in accordance with the 2007 Rwanda national ART treatment guidelines, which were in line with WHO recommendations at that time [22], [23]. First-line regimens included a combination of either two nucleoside-analogue reverse transcriptase inhibitors (NRTI) and one non-nucleoside reverse transcriptase inhibitor (NNRTI) or a combination of three NRTIs. Response to ART was routinely monitored on the basis of clinical symptoms and CD4 count as recommended by the national guidelines. HIV VL and HIVDR genotyping tests foreseen in the study design were not performed in real time and hence could not be used for the clinical management of study participants. However, the study clinicians could request additional VL and/or CD4 count testing if they suspected clinical or immunological failure to support the decision to switch to a second-line regimen. Results of retrospective VL and HIVDR genotypes were available after study completion and were reported to the study clinicians.

### Monitoring of drug adherence

ART adherence was captured in three different ways. First, a standardized questionnaire including questions on frequency of dosing, missed doses, drug sharing and reasons for poor adherence was administered by the study nurse at month 1 and at every three-month visit thereafter. Second, pill counts were recorded at each monthly pharmacy visit. Third, the attendance rates of medical and pharmacy appointments (with a 7 day window) were recorded. Participants were classified as fully adherent if they took more than 95% of their prescribed ART regimen doses and as poorly adherent otherwise.

### Blood sample collection and storage

Blood samples for laboratory testing were collected at baseline, month 6 (CD4 count only) and month 12. Five milliliters (mL) of whole blood was collected in EDTA vacutainer tubes (Becton Dickinson, Franklin Lakes, NJ). Fifty microliters (µl) of fresh blood was used for CD4 cell enumeration. Plasma was separated from the cellular fraction by centrifugation and collected into three aliquots of one mL each, within four hours after blood collection. Plasma samples were stored at −80°C until further analysis.

### Laboratory investigations

#### CD4 count

CD4 count was determined at baseline, month 6 and month 12 in whole blood on a single flow-cytometry platform using TruCOUNT® tubes on a FACScalibur instrument (Becton Dickinson, San Jose, CA, USA) and according to the manufacturer's instructions.

Immunological failure was defined as failure to achieve a CD4 gain of at least 50 cells above pre-therapy levels or having an absolute CD4 count of less than 100 cells/µl after one year of therapy [23].

#### HIV-1 viral load

HIV RNA VL was measured at baseline and month 12. Viral RNA quantification was performed on thawed plasma using the Roche CobasAmpliPrep/CobasTaqMan HIV-1 version 2 (Roche Molecular Systems, France) and according to the manufacturer's instructions. The lower limit of detection was 40 copies of HIV RNA/mL. Virological treatment failure was defined as a confirmed VL>1000 copies/mL at month 12.

#### HIV-1 DR genotyping

HIVDR genotyping was done retrospectively on samples from participants that had completed their month 12 visits and had available VL data at baseline and month 12. Baseline and month 12 samples with a VL>1000 RNA copies/mL were analyzed at the Department of Medical Microbiology (Academic Medical Centre, Amsterdam) using the Viroseq HIV genotyping kit version 2 (Abbot Molecular Inc, IL, USA) on an automated sequencer ABI 3130 XL (Applied Biosystems, Carlsbad, CA, USA). Sequences of amplified viral genes coding for the HIV-1 protease and reverse transcriptase enzymes were assembled, edited using the software provided by Viroseq, and submitted to the Stanford University database. Baseline sequences were categorized according to the WHO list of mutations for surveillance of transmitted drug resistant HIV strains (2009 update) [24]. Month 12 sequences were categorized according to the International AIDS Society-USA drug resistance mutations group (December 2010 list) [25]. HIV-1 subtypes were determined using the REGA HIV-1 Subtyping Tool (Version 2.0) available from the Los Alamos database [26] on the same gene sequences. The sequences generated in this study are available in the GenBank repository with accession numbers KC841660-KC841778.

### Statistical methods

Analyses were conducted with STATA version 11 (STATA Corporation, College Station, TX).

Baseline characteristics were reported as percentages for categorical data and means with standard deviations (SD) for continuous data. Differences between the groups were tested using the Pearson Chi-square test, the student's t-test and the Fisher's exact test, as appropriate. Stem and leaf plots and Shapiro-Wilks test were used to investigate the normality of data distribution. If variables deviated from the normal distribution, medians and interquartile ranges (IQRs) and non-parametric tests were used.

Potential risk factors for virological treatment failure at month 12 were examined by bivariable and multivariable logistic regression analysis. Factors were included in the multivariable analysis when p-values<0.2 in bivariable analysis [27]. Factors known to be associated with virological failure from the literature, such as age, baseline HIV viral load and treatment adherence were included in the multivariable model regardless of the strength of their association with VL in bivariable analysis [28]. Co-linearity was checked by performing a linear regression analysis instead of the logistic regression analysis to calculate the variance inflation factors, which were all below 2. Missing values were excluded from all analyses. The level of significance was set at p<0.05.

## Results

### Study profile

**Figure 1:**
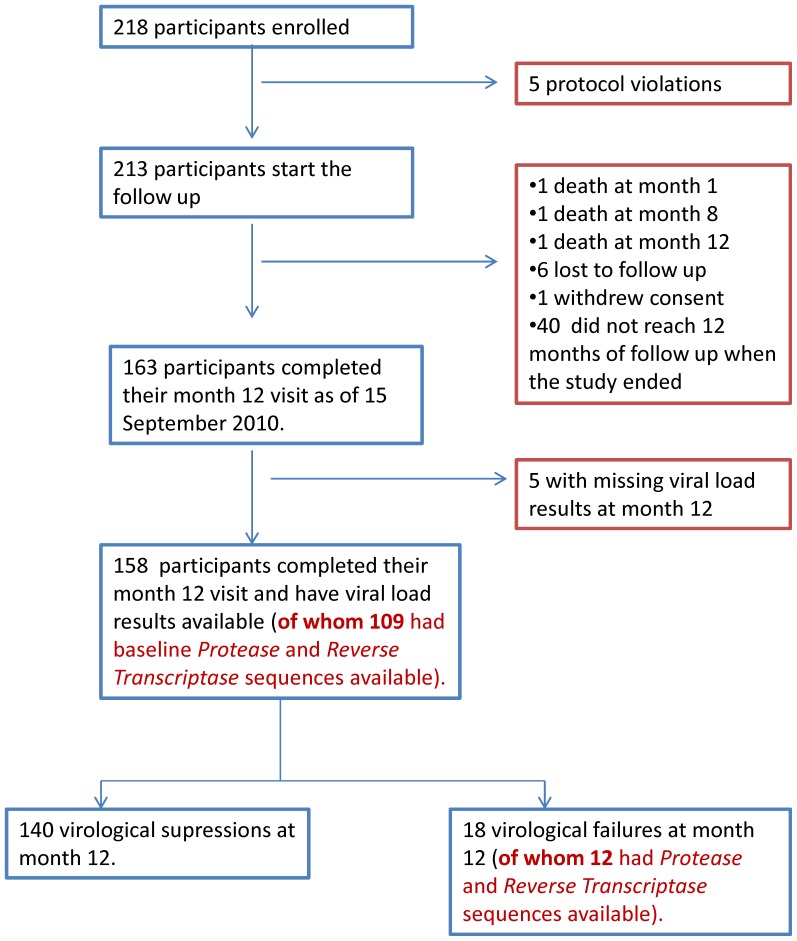
Study profile. Of the 218 were enrolled in the study, 213 started the 12 month follow-up. One hundred and fifty-eight participants completed their month 12 visit and had viral load results available at baseline and month 12. Of these 140 could be classified as virological successes (VL≤1000 copies/mL) and 18 as virological failures (VL>1000copies/mL).

Two hundred and eighteen HIV-1 positive participants (52.3% women) visited the TRAC-Plus clinic, were eligible for ART and consented to participate in the study (Figure 1). Of these, 5 participants were erroneously enrolled and were excluded from the analysis. Two hundred and thirteen participants were prospectively followed. One patient withdrew his consent. Three of 213 participants (1.4%) died during follow-up: two were reported to have committed suicide (at months 1 and 8) and the third participant discontinued ART after eight months due to social reasons and died from tuberculosis at month 12. Six of 213 participants were lost to follow-up (3%) while 40 active participants in the cohort had not reached 12 months of follow-up at study closure in September 2010. In total, 203 participants of the initial cohort of 213 (95.3%) had been retained in care at the end of the study period, of whom 163 had reached 12 months of follow-up. One hundred and fifty-eight participants had VL results available at month 12 and could be classified into 140 virological treatment successes and 18 treatment failures.

### Baseline characteristics of the study participants

At ART initiation, the 158 participants with 12 months of follow-up who were included in this analysis did not significantly differ from the 213 participants who enrolled in the SEARCH study for any of the baseline parameters collected (data not shown). The mean age was 37.9 years (SD = 7.6) and 55.1% were females (Table 1). Of the 158 participants included in the analysis, 52.0% were married 43.0% did not know their sexual partner's HIV status. Only 14.4% of the patients with a known HIV-positive partner were aware of their partner taking ART. Patients with and without virological failure at month 12 were comparable for all socio-demographic parameters (Table 1).

**Table 1:** Baseline characteristics of participants that received 12 months of ART.

Characteristics	Viral load testing at	Virological	Virological	p value
	month 12 N = 158	Suppression N = 140	failure N = 18	
	n(%)	n(%)	n(%)	
Age in years (mean, sd)	37.9(7.6)	38.3(7.4)	35.1(8.9)	0.09
**Gender**: Female	87(55.1)	77(55.0)	10(55.7)	0.96
**Education level**:				
None	12(7.8)	11(8.1)	1(5.6)	0.72
Primary	66(42.9)	58(42.6)	8(44.4)	
Secondary	68(44.2)	59(43.4)	9(50.0)	
Post-secondary	8(5.2)	8(5.9)	0(0)	
**Marital status:**				
Never married	15(9.9)	15(11.0)	0(0)	0.27
Married	79(52.0)	72(52.9)	7(43.8)	
Divorced	37(24.3)	32(23.5)	5(31.2)	
Widowed	21(13.8)	17(12.5)	4(25.0)	
**≥2 sex partners in the last year**	10(6.8)^1^	10(7.7)^2^	0(0)^3^	0.24
**Alcohol use** *	68(43.9)	57(41.6)	11(61.1)	0.12
**Condom use during the last sex act**	68(44.4)	63(46.3)	5(29.4)	0.20
**Age at sexual debut (median years, range)**	19(6–31)	20(6–31)	18(12–21)	0.06
**Partner HIV status:**				
Negative	20(12.8)	19(13.8)	1(5.6)	0.42
Positive	69(44.2)	62(44.9)	7(38.9)	
Unknown	67(43.0)	57(41.3)	10(55.6)	
**Partner on ART**	29(19.6)^4^	26(20.0)^5^	3(16.7)^6^	0.59
**Received ART for PMTCT among females**	14(17.5)^7^	14(19.4)^8^	0(0)^9^	0.32
**WHO stage**: Stage 1	93(60.0)	87(63.5)	6(33.3)	**0.01**
Stage 2	37(23.9)	32(23.4)	5(27.8)	
Stage 3	21(13.6)	16(11.7)	5(27.8)	
Stage 4	4(2.6)	2(1.4)	2(11.1)	
**Median CD4+ T cell count**	215(129–278)	219(139–272)	129(48–282)	**0.04**
**(cells/µl, IQR)**				
**Median HIV-1 viral load**	4.8(4.2–5.2)	4.8(4.2–5.2)	4.9(4.3–5.4)	0.32
**(Log_10_ RNA copies/mL, IQR)**				
**ART regimen:**				0.22
AZT+3TC+NVP/EFV	140(89.0)	126(90.0)	14(77.7)	
d4T+3TC+NVP/EFV	11(7.0)	8(5.7)	3(16.7)	
TDF+3TC+NVP/EFV	7(4.0)	6(4.3)	1(5.6)	
**Baseline HIVDR mutations**	4(3.6)^10^	1(1.1)^11^	3(18.8)^12^	**<0.001**

1 n = 147,

2 n = 130,

3 n = 17,

4 n = 148,

5 n = 130,

6 n = 18,

7 n = 81,

8 n = 72,

9 n = 9,

10 n = 109,

11 n = 91,

12 n = 16.

Statistical differences between virological treatment failures (n = 18) and virological treatment success after 12 months of ART were determined by student's t test for continuous normally distributed data, Wilcoxson rank sum test for non-parametric continuous data and chi-square and fisher's exact where appropriate for categorical data.

* drinking any quantity of alcohol at least 3 days a week, every week in the last 6 months.

All participants were prescribed an appropriate ART regimen as per the 2007 Rwanda ART National guidelines. A combination of zidovudine (AZT), lamivudine (3TC), and nevirapine or efavirenz (NVP/EFV) was the most commonly (89%) prescribed first-line regimen. The other first-line regimens contained tenofovir (TDF; 4%) or stavudine (d4T; 7%) instead of zidovudine. Women with a previous history of PMTCT all received NNRTI-based regimen. Participants with virological failure at month 12 were more likely to have initiated ART at a more advanced WHO stage 3 or 4 (38.9%) than participants with virological suppression at month 12 (13.4%; p = 0.01). Participants with virological treatment failure also had lower baseline CD4 count (median = 129 versus 219 cells/µL; p = 0.04) and a higher proportion of baseline HIVDR mutations (1.1% versus 18.8%; p = 0.001) than participants with virological suppression.

### Characteristics of participants with HIVDR mutations at baseline

HIVDR genotyping was performed in pre-ART plasma specimens from 109 participants and HIVDR mutations were identified in 4 of them (3.6%; 3 women and 1 man). NNRTI HIVDR mutations were detected in all 4 participants. The K103N mutation was the single NNRTI mutation detected in 3 of 4 patients, with one of them also harboring the M184V NRTI mutation (Table 2). One patient had a combination of A98G, Y181C and G190A mutations. None of the women with baseline HIVDR mutation(s) had a history of ART in the context of PMTCT. The 4 participants with HIVDR mutations at ART initiation received ART regimens that were only partially active. During the 12 months of follow-up, none of these patients was switched to a fully active first-line or second-line regimen.

**Table 2:** Baseline HIVDR mutations.

Participant's	ART regimen	Viral load	CD4 count	major mutations			HIV	Virological outcome
Codes	at baseline∧	at baseline	(cells/µL)				subtypes	at month 12
		(RNA copies/mL)						
				NRTI*	NNRTI **	PI		
1¥	AZT/3TC/**NVP**	68500	112	None	K103N	None	C	Viral suppression
2	AZT/3TC/**NVP**	8300	416	None	K103N	None	C	Viral failure
3	AZT/**3TC**/**NVP**	141000	60	M184V	K103N	None	A1/C	Viral failure
4	AZT/3TC/**EFV**	68500	48	None	A98A, Y181C,	None	A1	Viral failure
					G190A			

∧ None of the participants with drug resistance at baseline were switched to alternative first-line or second-line treatment during the 12 month follow-up. The treatment shown was initiated after the baseline HIVDR genotyping.

¥ participant 1 showed baseline mutations, but was virologically suppressed at 12 months (see Table 4);

* M184V/I cause high-level in vitro resistance to 3TC.

** K103N and K103KN, A98AG and Y181CY cause high-level resistance to NVP and EFV. G190A causes high level resistance to NVP and intermediate resistance to EFV.

The drugs in bold and underlined have a reduced sensitivity against the mutated viruses.

Viral suppression is defined by VL≥1000copies/mL.

The HIV-1 subtype distribution in the SEARCH cohort has been described elsewhere [30]. Briefly, the most predominant HIV subtype among the 109 participants was HIV subtype A1 (70.9%), followed by recombinant A1/C (19.8%), subtype C (5.3%) and subtype D (3.0%).

Among the 4 participants with baseline HIVDR, the observed proportions of subtype C (2/4), A1 (1/4) and A1/C (1/4) were significantly different than those observed in the 105 participants with no HIVDR mutation at baseline (Fisher's exact test p = 0.036).

### Month 12 treatment outcomes

#### Treatment substitutions and switches

Forty-one substitutions within the first-line, and two switches from first- to second-line regimens, occurred during the study period (Table 3). The most frequently reported reasons for single drug substitutions within the first-line were: compliance with the change of the Rwandan ART guidelines recommending a TDF backbone instead of an AZT or d4T-based backbone, hepatotoxicity, and compensation for stock-outs of specific drugs. The two participants that were switched from a first- to a second-line regimen presented with virological failure at month 12 (Table 4). Participant 7 had an initial drug substitution within the first-line due to alleged hepatotoxicity at month 3. EFV was replaced by abacavir (ABC) using an NRTI backbone of TDF and 3TC. At month 7, this same patient was switched to a second-line regimen containing TDF, 3TC, lopinavir/ritonavir (LPV/r) based on failure to reach a CD4 count >100cells/µl (from 9 cells/µl at baseline to 11 cells/µl at month 6). Participant 15 was switched from d4T/3TC/NVP to a second-line regimen containing TDF/3TC/LPN/r at month 4 based on worsening of his clinical condition and the recurrence of an oral Kaposi sarcoma. All the other participants remained on the same first-line regimen up to their month 12 visit.

**Table 3:** Treatment outcomes during 12 months of follow-up.

Outcome	Month 12	Baseline CD4	Baseline CD4	P value
	(n = 158)	count≤200 (n = 70)	count>200 (n = 81)	
	n, (%)	n, (%)	n, (%)	
***Treatment switches and substitutions***				
**Switches to second-line treatment**				
TDF+3TC+LPV/R	2(1.3)	2(2.9)	0	-
**Substitution within first-line treatment**				
AZT/d4T to TDF	26(63.4)	14(63.6)	9(56.3)	0.38
AZT to d4T	1(2.4)	0	1(6.3)	
NVP to EFV	10(24.4)	7(31.8)	3(18.8)	
EFV to NVP	1(2.4)	0	1(6.3)	
NVP/EFV to ABC	3(7.3)	1(4.5)	2(12.5)	
***Virological outcomes***				
**Virological suppressed (VL<1000 copies/mL, %)**	140 (88.6)	58(82.9)	75(92.6)	0.07
**Virological treatment failure (VL ≥1000copies/mL, %)**	18 (11.4)	12(17.1)	6(7.4)	
**HIVDR mutation at month 12**	8∧∧(5.3)	6(9.0)	2^±^ (11.1)	0.09
***Immunological criteria for treatment failure***				
**Immunological failure among virologically suppressed**	32(23.8)	12(20.7)	20(27.0)	0.40
**Immunological failure among virologically failed**	9/18(50)	5(41.7)	4(66.7)	0.32

∧∧ n = 152: 140 treatment successes+12 treatment failures with a HIVDR genotype available.

±n = 12 treatment failures with a HIVDR genotype available.

**Table 4:** Characteristics of participants with virological failure at month 12.

Participant	Viral load	CD4	Immunological	HIV-1	Major gene mutations			ART regimen	Switched
codes	(RNA copies/mL)	(cells/µL)	Failure	subtype	NRTI	NNRTI	PI		To 2^nd^ line
			(yes/no)						
**2** *	9390	386	Yes	C	M184V	K103N	None	AZT/**3TC/NVP**	No
**3** *	7940	105	Yes	A1/C	M184V	K103N	None	AZT/**3TC/NVP**	No
**4** *	8100	71	Yes	A1	D67N, K70R	A98G, K101Q	None	AZT/**3TC/NVP**	No
					M184V, K219Q	G190A			
**5**	5480	306	No	A1	M184V	K103N	None	AZT/**3TC/NVP**	No
**6**	29800	159	Yes	A1	M184V	K103N, V108I	None	AZT/**3TC/NVP**	No
						G190A			
**7** **	8020	70	Yes	A1	D67N, K70R	Y181I	None	TDF**/3TC**/LPV/r	Yes: month 7
					M184V				
**8**	7920	137	Yes	A1	None	K103N, Y181C	None	AZT/3TC/**NVP**	No
						G190A			
**9**	9260	211	No	A1/C	None	K103N, V106M	None	AZT/3TC/**NVP**	No
**10**	82000	246	Yes	A1	None	None	None	AZT/3TC/EFV	No
**11**	2380	194	No	A1	None	None	None	TDF/3TC/NVP	No
**12**	346000	190	Yes	A1	None	None	None	TDF/3TC/EFV	No
**13**	86300	210	Yes	A1	None	None	None	TDF/3TC/NVP	No
**14**	7850	242	Yes	A1	NA	NA	NA	TDF/3TC/NVP	No
**15**	808000	192	No	A1	NA	NA	NA	TDF/3TC/LPV/r	Yes: month 4
**16**	2500	181	No	D	NA	NA	NA	TDF/3TC/NVP	No
**17**	63500	449	No	NA	NA	NA	NA	AZT/3TC/NVP	No
**18**	113000	115	No	D	NA	NA	NA	AZT/3TC/NVP	No
**19**	1700	552	No	A1	NA	NA	NA	TDF/3TC/NVP	No

A98G reduces NVP susceptibility, Y181C causes high-level resistance to NVP, G190A causes high level resistance to NVP and intermediate resistance to EFV, M184V cause high-level in vitro resistance to 3TC, K103N causes high-level resistance to NVP, and EFV, D67N, K70R and K219Q cause resistance to AZT and d4T.

* These participants showed mutations at baseline (see table 2).

** Participant sample failed sequencing at baseline.

NA- Not applicable since these samples failed to sequence at both baseline and month 12.

The drugs in bold and underlined have a reduced sensitivity against the mutated viruses.

#### Virological outcomes and related immunological criteria

The proportion of patients with virological failure was higher, although not significantly so, in the group of participants that initiated ART at CD4 count ≤200 cell/µl as compared to the group that initiated therapy at CD4 count >200 cells/µl (12/70 versus 6/81 participants; p = 0.07). None of the women that had received NVP or AZT for PMTCT experienced virological treatment failure at month 12. Only nine of the 18 participants with virological failure (50%) also experienced immunological failure at month 12 and would have been correctly identified as failing their treatment (sensitivity = 50%). Conversely, 32 of 41 participants with virological suppression (23.8%) would have been misclassified as experiencing treatment failure based on immunological criteria at month 12 (specificity = 77%). Although the difference was not significant, acquired HIVDR mutations at month 12 were more frequent in the group of participants with baseline CD4 count ≤200 cells/µl, as compared to participants with baseline CD4 count >200 cells/µl (6/70 versus 2/81; p<0.09).

### Characteristics of participants with virological failure at month 12

Samples from 12 of the 18 participants with HIV VL>1000 copies/mL at month 12 were successfully genotyped. Eight participants harbored major mutations while no evidence of acquired HIVDR was found in 4 participants (Table 4). HIVDR genotyping could not be performed for 6 participants due to technical difficulties (one participant) or insufficient plasma volume (5 participants; Table 4). Three participants with HIVDR identified at month 12 had pre-existing HIVDR mutation(s) at baseline (tables 3 and 4), and 2 of them (participants 2 and 4) had acquired additional mutations during follow-up (Tables 2 and 4).

All cases of acquired HIVDR involved at last one NNRTI mutation, with the K103N mutation being the most frequently observed (6/8), followed by the G190A mutation (3/8) and the Y181I mutation (2/8). The K101Q, V108I and V106M mutations were each observed once as a single mutation (Table 4). Combined NNRTI and NRTI mutations were seen in 6 of 8 participants and involved M184V alone in 4 cases or M184V in combination with thymidine analogue mutations (TAMs) in 2 cases (table 4). The 2 TAMs cases were of pathway 2 and included the D67N, K70R and K219Q mutations for participant 4 and the D67N, K70R mutations for participant 7, respectively. Among the participants with virological failure at month 12 and a known HIV subtype, the proportions of subtypes A1 (12/17), C(1/18), D (2/17) and recombinant A1/C (2/17) was significantly different than the subtype distribution among patients with treatment success (Fisher's exact test p = 0.026).

Patients with virological failure at month 12 were not less adherent than the rest of the group regardless of the method used to measure adherence. Levels of antiretroviral treatment adherence in this cohort have been analyzed elsewhere [29].

### Factors associated with virological failure at month 12

Bivariable analyses indicated that treatment adherence by pill count (OR 2.25, 95% CI:0.81–0.62) and baseline viral load (OR 1.32, 95% CI 0.74–2.36) were not significant risk factors for virological failure at 12 months. HIV subtype was not a significant risk factor for virological failure at month 12 either when comparing subtype C to subtype A1(OR 3.65 (95% CI 0.34–39.09) and when comparing recombinant subtype A1/C to subtype A1 (OR 3.65 (95% CI 0.34–39.09).

In the multivariable model, participants with advanced HIV disease defined by WHO HIV clinical stage 3 and 4 were more than 5 times more likely to have virological failure compared to those with WHO HIV clinical stage 1 (OR 6.31: 95% CI; 1.43–27.83, p = 0.02). In addition, severe immunosuppression at ART initiation (CD4 count <50 cells/µl) was significantly associated with virological failure at month 12 (OR 10.99: 95% CI;1.86–64.91, p = 0.008, see Table 5). The CD4 count at month 6 was not an indicator of virological failure at month 12., However, the odds of having a virological failure at month 12 was 5 times higher in participants with CD4 count ≤200 cells/µl (95% CI: 1.8–14.1) when compared to the others.

**Table 5:** Factors associated with virological failure at month 12.

	Crude			*Adjusted		
	Odds Ratio	95% CI	p value	Odds Ratio	95% CI	p value
**Age (years)**	0.94	(0.88–1.01)	0.09	0.96	(0.89–1.04)	0.32
**Adherence (pill count**)	2.25	(0.81–6.22)	0.12	3.01	(0.91–9.99)	0.07
**WHO stage** 1	reference			reference		
2	2.27	(0.65–7.94)	0.2	1.86	(0.45–7.57)	0.38
3&4	5.64	(1.69–18.77)	**0.005**	6.31	(1.43–27.83)	**0.02**
**Baseline CD4 count** >200	reference					
**(cells/µL)** 50–200	1.68	(0.53–5.29)	0.37	1.77	(0.49–6.36)	0.38
<50	10.42	(2.45–44.36)	**0.002**	10.99	(1.86–64.91)	**0.008**
**Baseline viral load**						
**(Log_10_ RNA copies/mL)**	1.32	(0.74–2.36)	0.35	0.91	(0.49–1.66)	0.76

* Adjusted model includes age, baseline viral load, adherence, WHO stage and baseline CD4 count, n = 138 (6 participants missing adherence data, 1 participant missing age, 3 participants missing WHO stage data, 7 missing baseline CD4 count and 3 participants missing baseline viral load results). Adherence is used as a binary variable: adherence/non adherence.

## Discussion

To our knowledge, this is the first prospective study describing virological and HIVDR outcomes in a cohort of HIV-1 patients initiating first line therapy in Rwanda. The study achieved WHO-suggested targets of virological suppression in at least 70% of patients, and no more than 20% of patients lost to follow up, 12 months after ART initiation [5], [31]. These results may partly reflect the elite character of the cohort and the good performance of the TRAC-plus clinic and may not be fully representative of other public HIV clinics in Rwanda.

A significant proportion of patients in need of ART presented late to HIV care services (25% were already at WHO stage 3 and 4, and 44% had a CD4 count ≤200cells/µl). In addition, higher frequency of virological failure and acquired HIVDR mutations was associated with lower CD4 count at baseline. Initiation of ART at a more advanced stage of HIV disease is common in sub-Saharan Africa and negatively impacts HIV care and treatment program outcomes [19], [20], [21], [32]. Recent findings from Rwanda indicate that earlier ART initiation could be achieved by improving pre-ART retention and linking HIV screening to HIV care and treatment [33].

As previously documented, immunological criteria were poorly correlated with virological treatment failure [34]. Clinical decisions based on immunological criteria alone would have led to an unnecessary switch to second line therapy in one in four virologically suppressed participants and would have delayed a switch to second line therapy in 50% of those with virological failure. These results corroborate previous reports and confirm the added value of regular viral load monitoring to detect virological failure in a timely fashion [15], [34]. The appropriate use of targeted VL at month 12 would have increased the positive predictive value of detecting virological failure from 22 to 100% as compared to using immunological criteria alone. In addition, recent cost-effectiveness studies suggest that HIVDR genotyping at treatment failure could also have economical and clinical benefits in settings characterized by low CD4 count at treatment initiation and a relatively high frequency of wild-type viruses among patient failing therapy [35]. Genotyping of patients failing treatment at month 12 would have allowed for deferring costly second-line therapy in at least 4 participants with persistent wild-type virus infections and in whom HIV might be re-suppressed by improving ART adherence. In the SEARCH study, however, targeted VL monitoring was requested for only 9 of 41 participants who were suspected treatment failures at month 12, highlighting possible difficulties in interpreting CD4 counts or potential barriers to the utilization of VL testing by clinicians. Encouraging clinicians to use available laboratory-based monitoring methods to support clinical decision-making could contribute to improved quality of HIV care and reduced HIVDR. HIV sequencing capacity is currently being established at the National Reference Laboratory in Kigali. This would enable genotyping as part of ART monitoring and could improve the long term success of ART programs in Rwanda.

The 11% of participants that developed virological failure after 12 month of treatment is comparable to findings from 12 low- and middle-income countries of Asia and Africa, reporting an average of 9.4% patients experiencing treatment failure after one year [31]. Although pre-therapy drug resistance was more frequent in participants failing therapy, it was not associated with virological failure at 12 months in bivariable analysis, possibly due to the small number of cases. The low level of transmitted HIVDR in our cohort is comparable to findings from WHO-designed surveys in the region [8], [31], agrees with the relatively recent history of ART scale up in Rwanda [18], [36], and suggests that a change in first-line ART is not warranted in the near future.

Among the 18 participants failing therapy, at least four (22%) did not show any evidence of HIVDR mutations, indicating that they are failing therapy for reasons other than drug resistance. One reason might have been sub-optimal adherence although none of the treatment adherence measures collected in this study was identified as a predictor for treatment failure. Eight of the 12 study participants (66.6%) genotyped at treatment failure had evidence of HIVDR, which is comparable to the 63.7% reported in more than 2000 HIV patients initiating first-line ART using the WHO approach in Eastern Africa between 2006 and 2010 [31]. Our results indicate that reliable measurements of drug adherence are needed.

Overall, the NRTI and NNRTI transmitted and acquired mutation patterns that we identified were consistent with previous reports in similar settings [14], [37], [38]. The most frequent NRTI mutation (M184V) and NNRTI mutations (K103N, 190G and Y181C) described in our study are known to be common in cases of treatment failure [31]. They are associated with the use of 3TC, EFV and NVP, which have low genetic barriers towards resistance. The frequent association of M184V with at least one NNRTI resistance-associated mutation (6/10) is also in accordance with the results of other studies [39]. M184V causes resistance to 3TC and FTC, enhances the susceptibility to AZT, and delays the emergence of mutations associated with AZT and d4T such as TAMs [40], [41], [42].

In 2 of 4 participants with HIVDR mutations at baseline, exposure to a failing regimen during 12 months was associated with the accumulation of additional HIVDR mutations, including the emergence of TAMs, also in association with M184V. M184V and TAMs confer cross-resistance to NRTIs and their relatively high overall prevalence in this cohort may have consequences for second line treatment responses in Rwanda. NRTI cross-resistance has the potential to significantly reduce the activity of the NRTI backbone of standard second line regimens. More specifically, TAMs have the capacity to reduce the efficacy of TDF containing-NRTI backbones. Functional PI monotherapy will lower the barrier for PI resistance [43]. Although poor adherence cannot be completely ruled out, the reduced activity of the NRTI backbone might have contributed to continuing viral replication in the face of second line treatment, which was observed in the 2 participants that were switched early.

Although our findings may not be generalized to all HIV clinics in Rwanda, they indicate that efforts to minimize HIVDR are needed. These should include improved availability and utilization of VL-based monitoring of ART response, and evaluation of the potential added value of HIV genotyping at treatment failure. In addition, high quality patient support for treatment adherence as well as earlier initiation of therapy will contribute to protecting the efficacy of second line and subsequent therapy and improving overall treatment outcome.
